# Role of Intestinal Ultrasound for IBD Care: A Practical Approach

**DOI:** 10.3390/diagnostics14151639

**Published:** 2024-07-30

**Authors:** Joerg C. Hoffmann, Tobias Ungewitter

**Affiliations:** 1 Gastroenterologie am Herrngarten, Frankfurter Str. 3, 64293 Darmstadt, Germany; 2 Medizinische Klinik I, Vincentiuskrankenhaus, 76829 Landau, Germany

**Keywords:** imaging, Crohn’s disease, ulcerative colitis, standardised reporting, diagnostic algorithm, therapeutic algorithm

## Abstract

Intestinal ultrasound (IUS) has recently become the imaging technique of choice for patients with different types of intestinal inflammation. IUS has a high sensitivity, specificity, positive predictive value, and negative predictive value when diagnosing Crohn’s disease or ulcerative colitis. Further, it is now the preferred imaging modality for routine IBD reevaluations because of its non-invasiveness, cost-effectiveness, availability (at least in Europe), and reproducibility in all age groups. However, the clinical success of IUS requires IUS training for doctors and technicians who perform IUS with a standardised description of ultrasound findings of the terminal ileum and entire colon. Complications such as abscess formation, fistulae, and stenosis can be detected by either conventional IUS or contrast-enhanced ultrasound (CEUS). Lately, several disease activity scores have been proposed for Crohn’s disease, postoperative Crohn’s disease, and ulcerative colitis both in adults (including elderly) and in children. IUS was successfully used in randomised clinical trials in order to measure the treatment response. Therefore, IUS now plays a central role in clinical decision making.

## 1. Introduction

Inflammatory bowel diseases, namely Crohn’s disease (CD), ulcerative colitis (UC), and unclassified colitis (IBD-u), are chronic inflammatory disorders of the gastrointestinal tract. They affect any age group, including children and elderly patients with IBD [1,2,3,4,5]. Imaging techniques, particularly ileocolonoscopy, are required in order to establish the correct diagnosis, the disease localisation, its extent, and its activity. The subsequent clinical course is heterogeneous as exemplified by the IBSEN cohort. While some patients have a long silent period after an initial flare-up, others show a chronic remitting course or even a persistent, severe inflammatory course [6]. Treatment planning requires regular assessments of disease activity, detection of complications, and response to treatment using laboratory tests and imaging [7,8]. Treatment targets can be mucosal healing, transmural healing, and histological healing, as well as early detection of complications. Current international recommendations (STRIDE 2) name mucosal healing as a treatment target in both CD and UC. However, transmural healing was not consented as an additional target [9]. In the meantime, a systematic review showed that transmural healing predicts a better outcome in CD and is best monitored through the measurement of bowel wall thickness (BWT) [10]. While endoscopy is the best way to monitor mucosal healing, it is unable to measure BWT and is of limited use in detecting complications such as abscesses, fistulae, or small bowel strictures. Further, regular ileocolonoscopies are invasive, expensive, and unpopular among patients [11]. An assessment of BWT as well as the detection of complications can be accomplished using intestinal ultrasound (IUS) as well as magnetic resonance enterography (MRE) both in CD and UC [7,8,12,13,14]. The EFSUMB guidelines for gastrointestinal ultrasound recommend the use of linear, high-frequency probes (>5 MHz) to delineate all bowel layers [15]. While IUS is quick, cost-effective, and widely available, MRE is time consuming, expensive, and not always available. Therefore, this paper will review practical aspects of IUS in IBD. It is based on a broad literature search as well as the authors’ comprehensive experience in IUS as well as IBD care.

## 2. Materials and Methods

### 2.1. Literature Search and Keywords

A literature search from 1970 to 2024 was conducted using PUBMED, Embase, and the Cochrane library by one of us (JCH). The first search included the following terms: (“inflammatory bowel disease” or “IBD” or “Crohn’s disease” or “ulcerative colitis” or “unclassified colitis”) AND (“ultrasound” or “US” or “ultrasonography”). A second search was conducted using the following terms: (“inflammatory bowel disease” or “IBD” or “Crohn’s disease” or “ulcerative colitis” or “unclassified colitis”) AND (“stricture” or “stenosis”) AND (“magnetic resonance enterography” or “MRE” or “CTE” or “ultrasound” or “ultrasonography” or “US” or “imaging” or “histology”). A third search included the following terms: (“inflammatory bowel disease” or “IBD” or “Crohn’s disease” or “ulcerative colitis” or “unclassified colitis”) AND (“ultrasound” or “ultrasonography” or “US” or “imaging”) AND (“clinical decision making” or “learning” or “cost savings”). The terms (“inflammatory bowel disease” or “IBD” or “Crohn’s disease” or “ulcerative colitis” or “unclassified colitis”) AND (“ultrasound” or “ultrasonography” or “US” or “imaging”) AND (“children” or “pediatric” or “elderly”) were used for the fourth search. All four literature search strategies were supplemented by a manual literature search.

### 2.2. Literature Selection

Publications from the above-mentioned literature search were screened based on the title and abstract. Studies, systematic reviews, and guidelines were selected based on clinical relevance regarding diagnosis, treatment monitoring, detection of complications, clinical decision making, and healthcare economics. All selected papers were further studied as full text manuscripts looking at classical ultrasound techniques as well as additional techniques (CEUS, elastography, and small intestinal contrast ultrasonography).

### 2.3. Image Acquisition

Ultrasound images were taken by two authors (JCH and TU) between 2016 and 2024 either at the St. Marienkrankenhaus, Ludwigshafen, Germany, or the practice Gastroenterologie am Herrngarten, Darmstadt, Germany, using one of the following ultrasound machines: Canon Aplio 400 employing the linear probe 11L5 (4.8 to 11 MHz), Canon Aplio i800 employing the convex matrix probe i8CX1 (1.6–8 MHz, used at 8 MHz) as well as the linear matrix probe i18LX5 (5.0–18 MHz), or GE Versana premier employing the linear probe L3-12RS (3.6–12 MHz).

## 3. Technique of IUS and Typical Findings in IBD

IUS in patients with either suspected or known IBD has been described in detail by the EFSUMB and others [7,15,16,17]. IUS of the small and large bowel can be accomplished using ultrasound scanners of at least a medium quality employing both a lower-frequency convex array probe (3–8 MHz) for general evaluation and a higher-frequency linear array probe (5–18 MHz) for high resolution with the possibility of an additional colour Doppler examination.

**Figure 1:**
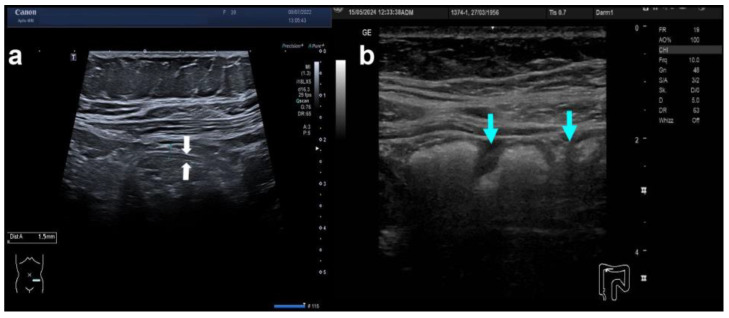
Intestinal ultrasound: normal colon. The transverse (**a**) and longitudinal (**b**) section of a normal colonic wall are shown. On the transverse section (**a**), the white arrows show the inner and outer margin with five layers in between (the white interface, the dark mucosa, the white submucosa, the dark muscularis propria, and the white serosa as the outer layer). On the longitudinal section (**b**), the normal haustration is indicated by the turquoise arrows.

Although fasting prior to IUSIUS has never been shown to enhance the imaging quality of bowel wall layers, the EFSUMB guideline recommends at least 4 h fasting similar to most studies on IUS in IBD [15]. The rationale is to improve the visibility particularly of small bowel loops. Most guidelines and reviews recommend scanning of the whole large bowel as well as the terminal ileum sequentially with the convex and then the linear array probe in two planes each. However, high resolution is mainly achieved with the linear probe starting at the terminal ileum and ending at the sigmoid colon. Therefore, we initially perform a general abdominal scan including solid organs plus the rectum (ideally with a full bladder) and then switch to the linear probe in order to thoroughly scan the whole ileocolon from the terminal ileum to the distal sigmoid colon. Others have advocated to scan the colon starting at the sigmoid colon and advance up to the coecum and the terminal ileum [7,17]. Sufficient compression and focus adaption are required to delineate all layers of at least the anterior part of the intestinal wall (Figure 1). The probe should be held perpendicular to the intestinal wall, particularly when measuring bowel wall thickness (BWT). While many authors advocate a complete transverse and longitudinal scan, we prefer a transverse plane for the whole ileocolon supplemented with longitudinal planes for each anatomical segment in order to evaluate haustration. An assessment of the small bowel can either be performed by scanning overlapping parallel lanes cranially and caudally or extensive small bowel ultrasonography of each loop after preparation with iso-osmolar polyethylene glycol (PEG) solution, a technique called small intestine contrast ultrasound (SICUS) [18]. A systematic approach is strongly recommended for IUS of both the large and small bowel [15].

**Figure 2:**
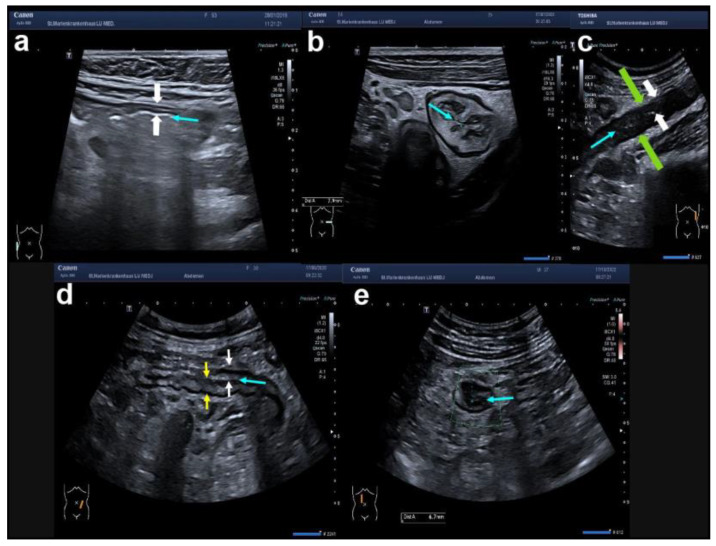
Variants of bowel wall stratification (BWS) and bowel wall thickness (BWT) in IBD: (**a**) normal BWS on a longitudinal colonic wall section with normal BWT (white arrows). The lumen is indicated by the turquoise arrow; (**b**) preserved layers with increased BWT due to a markedly thickened submucosa in a transverse section of the colonic wall; (**c**) complete loss of BWS in a longitudinal section of the descending colon. White arrows indicate the BWT and green arrows the total bowel diameter; (**d**) variants of BWS in a longitudinal section of a colonic wall (yellow: blurred BWS, white: preserved BWS); (**e**) asymmetric BWS in a transverse section of a transverse colonic wall.

During IUS, all bowel layers need to be visualised as shown in Figure 1 with normal haustration in the longitudinal plane. Seen from the lumen, the first layer is the hyperechoic interface between the lumen and the mucosa, which appears as a hypoechoic second layer. The third layer is again hyperechoic and represents the submucosa followed by the hypoechoic muscularis propria. The hyperechoic fifth layer represents the interface between the muscularis propria and the serosa. The total bowel wall thickness (BWT) is defined by the perpendicular distance between the inner part of layer 1 up to the outer part of layer 5 as shown in Figure 1. A normal BWT of the large intestine and small intestine is 3 mm or less. The normal wall of the sigmoid colon may reach 4 mm and the small bowel can already be inflamed at 3 mm. The bowel wall stratification should also be described with normal bowel wall stratification (BWS) in the normal intestine in contrast to altered BWS in CD and UC. Since CD is characterised by transmural inflammation and UC by only mucosal inflammation, one would assume that the alteration of either only the mucosa (UC) or all layers (CD) could help to distinguish CD from UC. However, severe acute inflammation usually leads to a complete loss of stratification in both CD and UC. Chronic inflammation can either result in an increased BWT with preserved BWS, blurred BWS, or asymmetric BWS, as shown in Figure 2. Spiculates are hyperechoic bands extending from the submucosa towards the mesentery indicating fibrosis in CD [19]. The measurement of BWT should be performed on the most inflamed part of each anatomical segment.

**Figure 3:**
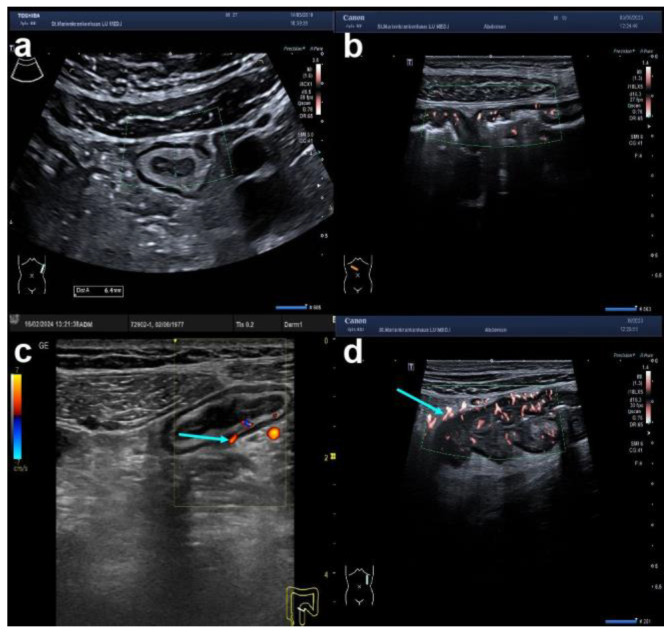
Variants of bowel wall vascularisation in IBD using colour Doppler signalling of colonic walls. Shown are typical examples employing the modified Limberg classification: (**a**) transverse section of the descending colon showing preserved layers with increased BWT but no signals on CDS employing superb microvascular imaging (SMI) (Limberg 1); (**b**) longitudinal section of the right transverse colon with orange dots within the blurred intestinal wall using SMI (Limberg 2); (**c**) oblique section of the sigmoid colon with preserved BWS, increased BWT and stretches of blood vessels within the intestinal wall using colour Doppler signalling indicated by the turquoise arrow (Limberg 3); (**d**) longitudinal section of the descending colon with markedly increased vascularity in the intestinal wall extending into the pericolonic echoenhanced tissue (turquoise arrow) using SMI (Limberg 4).

**Figure 4:**
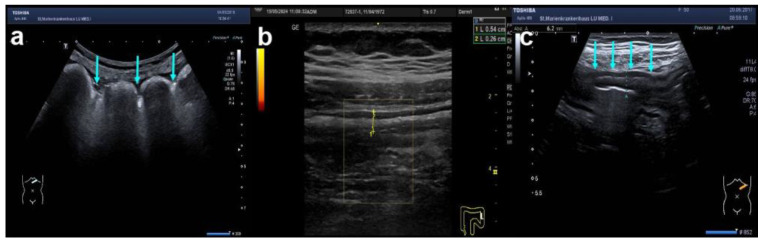
Variants of colonic wall haustration in IBD using B-mode ultrasound. Typical examples are shown as follows: (**a**) longitudinal section of the left transverse colon showing preserved haustration (turquoise arrows); (**b**) longitudinal section of the descending colon with increased BWT (yellow markers, number 1), preserved BWS, thickened submucosa (yellow markers, number 2), no hypervascularity (Limberg 1), and complete loss of haustration; (**c**) longitudinal section of the left transverse colon with increased BWT, thickened mucosa, and reduced haustration (turquoise arrows).

The third important parameter with regard to inflammation is vascularisation as shown in Figure 3 [20,21]. In order to detect low blood flow, the velocity range for colour Doppler investigations should be set to between 2 and 5 cm/sec. Using this setting, colour Doppler signals (CDSs) are only detected in inflamed bowel loops independent of its cause. Other modes like the power Doppler mode, advanced dynamic flow (ADF), or superb microvascular imaging (SMI) can be used alternatively with the advantage of being less angle-dependent. The following semiquantitative description by Limberg et al. is most commonly used for CDS: no signals but increased BWT is called Limberg 1, dots within the wall is called Limberg 2, streaks within the wall is called Limberg 3, and streaks within the wall extending into the peri-intestinal tissue is called Limberg 4 (see Figure 4 and Table 1) [21,22]. A modification of the Limberg classification has been used by others where no flow is called CDS 0 (irrespective of BWT), dots within the wall is called CDS 1 (equivalent to Limberg 2), streaks within the wall is called CDS 2 (equivalent to Limberg 3), and streaks beyond the wall is called CDS 3 (equivalent to Limberg 4) [23].

Apart from BWT, BWS, and vascularisation, IUS should be used to characterise the extent of bowel involvement. Characteristic patterns of either CD (e.g., segmental colitis or terminal ileitis) or UC (e.g., continuous inflammation) can indicate the diagnosis of either CD or UC. Further, loss of haustration (see Figure 4) is often found in UC, but it also occurs in Crohn’s colitis. Therefore, a definite distinction between CD and UC solely by IUS looking at disease localisation, haustration, specific complications (e.g., fistulae), and alterations of certain layers is often not possible. The same, however, applies to ileocolonoscopy and MRE [24].

**Figure 5:**
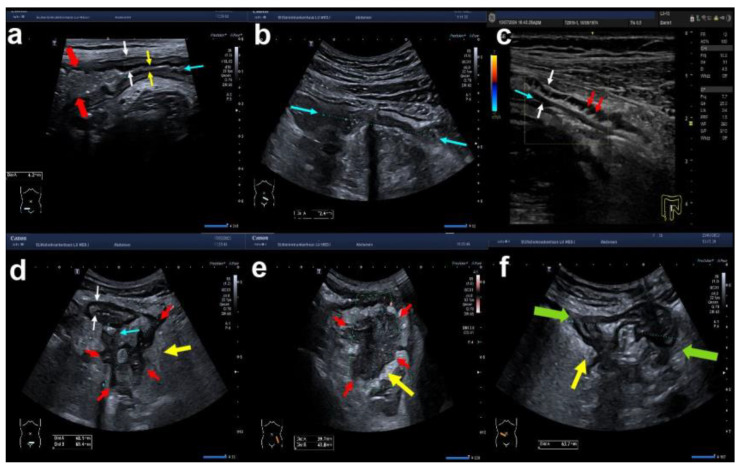
Variants of intestinal and extraintestinal complications of Crohn’s disease. (**a**) Longitudinal section of the terminal ileum in Crohn’s disease. Shown is a stricture with prestenotic dilatation (red arrows), luminal narrowing (yellow arrows), and increased BWT (white arrows). (**b**) Longitudinal section of a 7.2 cm long ileal stricture. (**c**) Intestinal fibrosis indicated by spiculates extending from the submucosa towards the serosa (red arrows) with increased BWT (white arrows); the lumen is indicated by the turquoise arrow. (**d**) A fistula (indicated by the turquoise arrow) extends from the sigmoid colon to an underlying abscess, which is shown by the red arrows. Fibrofatty proliferation is indicated by the yellow arrow leading to increased echogenicity. (**e**) B-mode image of an extra-intestinal abscess indicated by the red arrows. The yellow arrow shows the hyperechogenic surrounding inflammation. (**f**) Adherent, inflammatory small bowel loops form a pseudotumor (green arrows) with surrounding fat wrapping (yellow arrow).

If strictures are present, they should be described in detail. This includes the length of the inflamed bowel segment, the smallest luminal diameter of each stricture, and the presence or absence of small bowel dilatation (luminal diameter > 25 mm) (see Figure 5a,b). Markers of fibrosis are spiculates which appear as hyperechogenic bands extending from the submucosa towards the serosa as demonstrated in Figure 5c [19]. Additionally, complications of CD such as fistulae, phlegmons, abscesses, and inflammatory pseudotumours can usually be detected by IUS. Typical findings are shown in Figure 5d–f. Sometimes, the appearance of a phlegmon, an inflammatory pseudotumour due to adherent, inflamed small bowel loops, can be similar to either fistulae and/or abscesses on a B-mode scan. The colour Doppler mode can be helpful. However, contrast-enhanced ultrasound (CEUS) using the echo-signal enhancer sulfur hexafluoride (SonoVue^®^) is even better in differentiating perfused versus non-perfused tissue, i.e., phlegmons (perfused) versus abscesses or fistula tracts (non-perfused) [25]. Between 2.4 and 2.8 mL of SonoVue^®^ is usually required for CEUS using the linear higher-frequency probe while 1.2 to 1.4 mL are sufficient when using the convex lower-frequency probe. 

**Figure 6:**
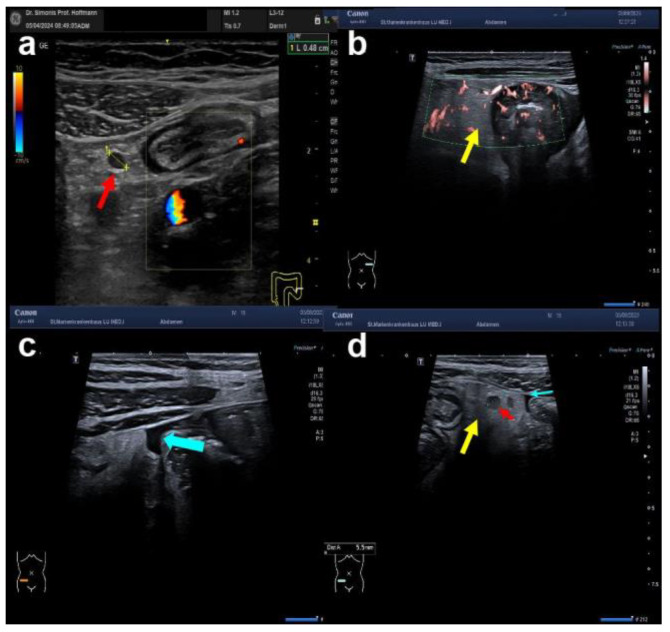
Additional extraintestinal ultrasound findings in IBD. (**a**) Shown is a round hypoechoic lymph node next to the thickened wall of the descending colon in hyperechoic tissue (fat wrapping) (yellow arrow). (**b**) The tissue surrounding the thickened strongly hypervascularised colonic wall (Limberg 4) is hyperechoic known as fat stranding, fat wrapping, or inflammatory fat (i-fat) (yellow arrow). (**c**) Ascites shown as an anechoic area indicated by a turquoise arrow. (**d**) Patient with Crohn’s disease showing all three additional extraintestinal signs, i.e., fat wrapping (yellow arrow), pericolonic lymph node (red arrow), and small amount of ascites (turquoise arrow).

Further, the following extraintestinal characteristics of IBD should be looked for: enlarged lymph nodes, small amounts of ascites (CD and UC), and inflammatory fat in the mesentery (iFAT or fat wrapping) appearing hyperechogenic (examples shown in Figure 6).

The above-mentioned techniques have been implicated in standard IUS. A standardised ultrasound report of IUS is recommended for all patients with suspected and known IBD. An example of a standardised report is shown in Table 1.

An ultrasound research tool aiming at a more sensitive detection of disease activity is again CEUS [26]. However, quantification of peak enhancement, wash-in and wash-out phases is limited to research facilities and measurements cannot be compared between different devices [27]. Regarding stricture assessment, the distinction between inflammation and fibrosis can also be addressed by using elastography. Although a systematic review suggests a moderate to good correlation between elasticity and fibrosis [28] a more recent study did not find real-time elastography to be reproducible [19]. Therefore, as of today, these special techniques do not play a role in standard IBD care.

## 4. Diagnostic Value of IUS in IBD and Comparison with Other Sectional Imaging Techniques

IUS can be used for the primary evaluation of patients with suspected CD or UC. It can also be applied to follow-up evaluations once CD or UC has been diagnosed. Further, IUS helps to quantify disease activity as well as treatment response and aids in the detection of complications. Overall, IUS has recently been shown to play an important role in IBD clinical decision making both in children and adults [29,30]. The most recent systematic review on adults reported a pooled diagnostic accuracy of bowel ultrasound in IBD of 66%, a sensitivity of 88.6%, a specificity of 86%, a positive predictive value of 94%, and a negative predictive value of 74% in adults when compared to endoscopy, histology, and/or intraoperative findings [31]. With regard to small bowel CD, the sensitivity was even higher for SICUS (97%). These data confirm a previous systematic review from 2014 [32]. A systematic review on IUS in the paediatric population showed slightly lower values than in adults (sensitivity of 84.1%, specificity of 82.9%) [33].

Multiple studies have compared IUS with other imaging techniques. Panes et al. published a systematic review in 2011 comparing IUS with MR and CT enterography (MRE, CTE) [34]. Although the sensitivity of MRE was found to be slightly higher (93%) than IUS and CTE (84 and 81%, respectively) the specificity of IUS was the highest at 92% (MRE: 90%, CTE: 88%). Radiation exposure must be considered when performing a CTE even with special protocols and, therefore, CTE is now only recommended for emergency situations (e.g., sepsis or suspected perforation) usually after either IUS or MRE. When looking at children, the pooled sensitivity and specificity of MRE was higher than for IUS (sensitivity 93 vs. 84.1%, specificity 94.6 vs. 82.9%) [33]. More recent studies, however, reported almost equal values in monocentric studies comparing IUS with MRE [12,35]. Importantly, interobserver reliability was found to be similar when looking at IUS and at MRE in CD [36,37] and also in UC [38]. With regard to different parts of the intestinal tract, high values (accuracy, sensitivity, and specificity) were reported for MRE-based disease assessment both in the small and large intestine when compared to ileocolonoscopy [39].

## 5. Role of IUS for Quantification of IBD-Disease Activity, Treatment Monitoring, and Outcome Prediction

Quantification of disease activity is important for risk stratification and treatment monitoring in both CD and UC. As mentioned above, the current international guideline STRIDE 2 recommends not only clinical remission but also mucosal healing as a treatment target [9]. Further, transmural healing and, most recently, histological healing have been shown to predict a better long-term outcome [10,40]. To this end, a widely available, safe, affordable, and precise imaging technology is needed for modern IBD care, particularly for primary risk stratification and even more important for follow-up evaluations. While ileocolonoscopy (ICS) is clearly required for the initial diagnosis, regular ICSs are problematic because of their invasiveness, limited availability, and high cost. Further, ICS can only assess the mucosa but not the whole bowel wall, i.e., transmural healing. Although MRE can address the whole bowel wall, it is also not the preferred diagnostic tool for treatment monitoring because of limited availability and high costs. To this end, IUS seems to be the ideal imaging modality for treatment monitoring. And indeed, several studies have looked at IUS as follow-up imaging during IBD treatment, both in CD and, more recently, in UC [38,41,42,43]. In order to quantify disease activity, multiple scores for adults have been published and validated as summarised in a systematic review by Goodsall [44]. The most widely used ultrasound activity scores that have been validated are the simple IUS score [45,46], the bowel US score (BUSS) [47], the international bowel ultrasound segmental activity score (IBUS-SAS) for CD [23,48], and the Milan US criteria (MUC) [49,50] as well as the UC IUS index for UC [51,52]. More recently, scores were also reported and validated for disease activity in children [52,53].

Ultrasound studies on IBD treatment response were systematically reviewed in 2022 and 2024 [54,55]. For instance, the IBUS-SAS was used in the STARDUST multicentre randomised controlled trial [43] and the UC IUS index in a monocentric prospective trial [38]. Therefore, IUS is increasingly used to objectify disease activity in clinical trials. Using such activity indices in routine IBD care can, however, not be recommended at this point.

Sonographic remission was also shown to predict better outcome in several studies on patients with CD [56,57,58,59,60] and more recently in one retrospective study on patients with UC [61]. Prediction of CD treatment response can be seen as early as 4 weeks after the start of anti-TNF-α monoclonal antibodies [62].

## 6. IUS-Based Detection of Fistulae, Inflammatory Masses, and Abscesses in Patients with Crohn’s Disease

Detection of inflammatory masses and abscesses by ultrasound were first reported in 1983 by Yeh and Rabinowitz and shortly after by Worlicek [63,64]. The first IUS report of CD patients with fistulae was published in 1996 including the B-mode features of abscesses [65]. The same group showed that CDS using the power mode is helpful for differentiating abscesses from inflammatory pseudotumours in CD patients [66]. Today CEUS is the preferred imaging modality used to prove the characteristic lack of vascularisation in an abscess or fistula [25]. The overall diagnostic accuracy of IUS regarding CD intraabdominal complications was recently summarised in a systematic review by Pruijt, showing sensitivities of above 80% and specificities over 90% [67].

## 7. IUS-Based Characterisation of Different Forms of Crohn’s Disease Strictures

Apart from penetrating complications (i.e., fistulae, inflammatory masses, and abscesses), CD can lead to strictures. Frequently, they are localised in the small bowel. They can be detected by endoscopy, CTE, MRE, and IUS. While all strictures lead to abdominal pain and obstruction (stenosis plus prestenotic dilatation) it is important for treatment planning to characterise the type, location, and length of strictures. Strictures can be classified into inflammatory strictures, fibrotic strictures, neoplastic strictures, and complex strictures (with fistulae). Although the detection of strictures can usually be achieved by ICS, CTE, MRE, or IUS, the distinction between inflammation and fibrosis is sometimes difficult [68]. In every patient, endoscopy is recommended in order to diagnose the occasional neoplastic stricture [69]. IUS has no role in cancer surveillance of patients with UC or Crohn’s colitis since intraepithelial neoplasia/dysplasia can only be detected by endoscopy and biopsies [69].

To define the best possible imaging modality for various non-neoplastic forms of strictures, it is important to look at their histopathology [70]. Recent studies show that CD strictures very often result from muscular hypertrophy and not only inflammation and/or fibrosis [71]. In addition, CD strictures can contain fat accumulation, which is seen on CT scans as the so called “fat halo sign” [72]. The most important clinical question, however, is whether a stricture can be targeted by a pharmaceutical approach and whether this can be predicted by an imaging modality [70]. So far, neither fat accumulation, fibrosis, nor muscular hypertrophy can be addressed with any medication [68]. To this end, imaging must select those patients who have inflammation in the stricture. The first study that compared the echo structure of the affected intestinal wall with the postoperative histopathology suggested that fibrosis was characterised by the combination of increased BWT and preserved BWS [73]. Loss of BWS with a hypoechoic echo pattern was mostly found in strongly inflamed strictures. Later studies looked at sonographic findings of strictures and their histopathology in more detail [19,74,75]. These studies suggested that certain echo patterns cannot distinguish between acute inflammation and fibrosis. For example, increased mucosal thickness can be due to acute inflammation and/or chronic inflammation and/or fibrosis, and increased thickness of the submucosa can be due to both acute and chronic inflammation. Surprisingly, increased mucosal thickness correlated in one study best with fibrosis [74]. Peri-intestinal inflammatory fat (iFAT) was mainly found in acute or chronic inflammation. Blurred or even lost BWS was seen in all types of strictures, showing the highest correlation with fibrosis. These interesting results demonstrate the challenges in interpreting sonographic findings as they relate to the underlying histopathology. The most recent analysis of strictures was published by Allocca et al. with two important findings. First, when spiculates are detected, this strongly indicates fibrosis (see Figure 5f). Second, hypervascularisation (Limberg 3 and 4, see Figure 3c,d) was significantly associated with active inflammation [19] confirming previous findings with CEUS and Doppler by Nyland et al. and Rippoles et al. [25,76]. A systematic review, therefore, concludes that “the capability of IUS for characterising inflammation versus fibrosis in strictures is not accurate enough to be used in clinical practice” [77].

## 8. IUS in Postoperative Care of Crohn’s Disease and in IBD Care during Pregnancy

Although intestinal resection of CD patients decreased up to 50% due to recent therapeutic advances, every other CD patient still needs surgery [78]. Postoperative management requires the detection of recurrence, which can be performed using either endoscopy or imaging, i.e., MRE or IUS. While ICS is the gold standard, the detection of CD recurrence via imaging techniques can reduce the number of ICSs, provided that these techniques have a high sensitivity and specificity. In 2018, two systematic reviews concluded that IUS has a high sensitivity and specificity in predicting recurrence (94% and 84%, respectively) [79,80]. Importantly, the specificity was slightly higher when comparing IUS to MRE (86% vs. 84%). The most recent systematic review showed a specificity for MRE of 78% [81]. Special ultrasound techniques such as SICUS or CEUS can increase the sensitivity while reducing specificity [79]. Because of the reduced specificity and problems with quantification in CEUS, they are not recommended for use in routine clinical practice.

Apart from postoperative management, the IBD care of pregnant women with either CD or UC is a special topic. Several groups have reported on their IUS experience in prospective observational studies [82,83,84]. It was shown that IUS is feasible and accurate throughout pregnancy, particularly in the first and second trimester. Even in the third trimester, the specificity was excellent with a somewhat reduced sensitivity (66.6%).

## 9. Role of IUS in IBD Clinical Decision Making

**Figure 7:**
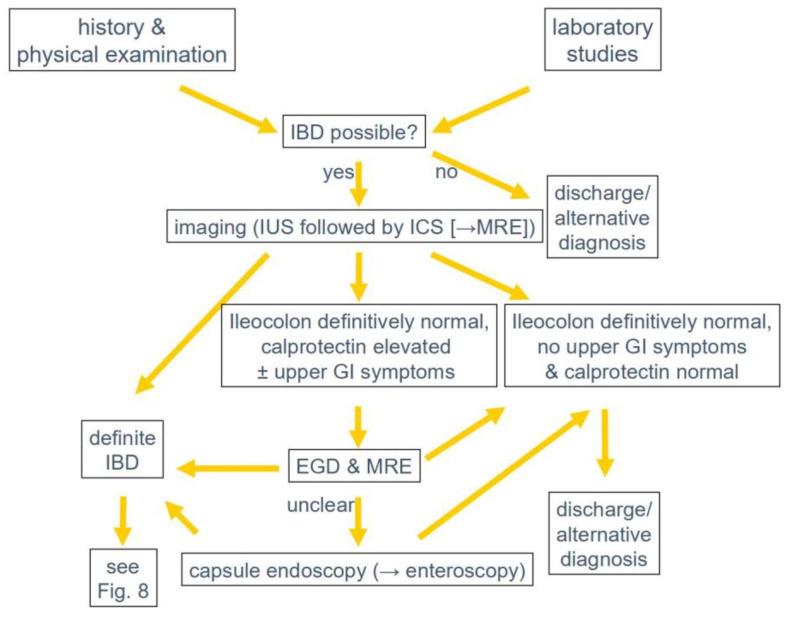
Diagnostic algorithm in suspected inflammatory bowel disease (IBD). EGD = esophagogastroduodenoscopy, IUS = intestinal ultrasound, ICS = ileocolonoscopy, MRE = magnetic resonance enterography.

**Figure 8:**
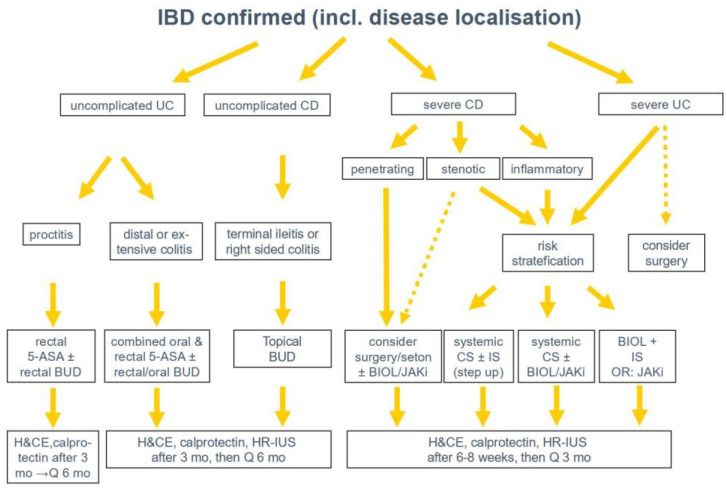
IUS-based therapeutic and follow-up algorithm. IBD = inflammatory bowel disease; UC = ulcerative colitis; CD = Crohn’s disease; ASA = amino-salicylic-acid; IUS = high resolution intestinal ultrasound; BUD = budesonide; BIOL = biologicals; JAKi = JAK inhibitor; CS = corticosteroids; IS = immunosuppressants, i.e., azathioprin; mo = months; H&CE = history and clinical examination; Q = every.

Over recent years, IUS has become part of the standard IBD care almost everywhere, sometimes as an early diagnostic modality but particularly as an imaging modality during follow-up [85]. Clearly, it has an important role regarding the detection of other entities as differential diagnosis at first presentation or during follow-up [86]. Apart from history and clinical examination, IUS together with calprotectin is now the cornerstone of IBD follow-up visits [87,88]. The reason for this development is the increasing evidence that IUS can guide therapeutic management [88,89]. Today, it can be used in out- and inpatient settings including specialised IBD flare clinics [30,61,87,90]. Its role is well shown for establishing the initial diagnosis, measurement of disease activity, detection of complications such as strictures, and penetrating CD complications, as well as the monitoring of treatment response or detection of postoperative recurrence. A diagnostic algorithm for suspected IBD is shown in Figure 7. An IUS-based treatment and follow-up algorithm is outlined in Figure 8. A health-economic study showed that an IUS-based management pathway is both quicker and cheaper than an MRE-based pathway [91].

## 10. Conclusions

Clinical IBD research has revolutionised IBD diagnostics and treatment. IUS became the imaging modality of choice for routine follow-up visits because of its high diagnostic accuracy, high reproducibility, wide availability, and limited costs. To this end, IUS together with calprotectin and standardised history taking and examination is now the best IBD standard of care for routine follow-up evaluations [6,88]. Certain limits (e.g., difficult anatomical areas such as the small pelvic region and jejunum), severely obese patients, and certain CD complications (e.g., the detection of fistulae) need to be kept in mind. In these situations, other imaging modalities, particularly MRE and endoscopy, have to be initiated. Endoscopy is also the examination of choice for IBD cancer surveillance. Since ultrasound performance is not a part of all gastroenterology training programmes, it is of utmost importance that training in abdominal ultrasound and particularly IUS is strongly promoted. Training programmes have started and it has been shown that basic competence in IUS can be acquired with relatively few examinations [17,92,93].

## Figures and Tables

**Table 1:** Standardised ultrasound reporting for suspected or known inflammatory bowel disease.

Category	Parameter	Subgroup	Detailed Description
General	Bowel visualisation	rectum	[complete; incomplete; not visualised]
		jejunum	[complete; incomplete; not visualised]
		ileum	[complete; incomplete; not visualised]
		coecum and ascending colon	[complete; incomplete; not visualised]
		transverse colon	[complete; incomplete; not visualised]
		descending colon	[complete; incomplete; not visualised]
		sigmoid colon	[complete; incomplete; not visualised]
	Local tenderness		[yes; no; unclear]
Bowel wall	Bowel wall thickness (BWT)		[in mm (>3 mm without increased vascularity: Limberg 1)]
	Submucosa thickness (optional)		[in mm]
	Bowel wall stratification (BWS)		[preserved stratification; blurred stratification; localised loss of stratification; generalised loss of stratification]
	Submucosal spiculates		[present; absent]
	colour Doppler signal (CDS)	vascularity	[normal; increased within the wall (dots, Limberg 2); increased within the wall (streaks, Limberg 3); increased within the wall extending into the surrounding mesentery (streaks, Limberg 4)]
Luminal changes	Small bowel dilatation		[present; absent (lumen diameter > 25 mm)]
	Luminal narrowing (small bowel)		[present; absent (lumen diameter < 10 mm)]
	Stricture length	(small bowel luminal narrowing)	[present (in mm for luminal narrowing); absent]
Surrounding	Peri-intestinal fat (creeping fat)	(inflammatory fat or iFAT)	[normal; slightly increased hyperechoic tissue; markedly increased hyperechoic tissue]
	Mesenteric lymph nodes		[not visible; visible, normal; visible, enlarged, reactive; visible, enlarged, likely neoplastic]
	Fluid (hypoechoic or echofree)		[little interenteric (<20 mm); large, localised (>2 cm); free ascites]
	Air (hyperechoic dot, extramural)		[outside the wall; within a fistula; within a localised fluid collection (abscess); within free fluid (free perforation)]
	Special features	Inflammatory tumour/mass	[present; absent]
		abscess (hypoechoic area, no CDS)	[present; absent]
		fistula (hypoechoic tract)	[present; absent]
		Others	[present; absent]
Summary: Segmental Crohn’s colitis (ascending and descending colon), maximum BWT of 11 mm, in the descending colon, loss of stratification, CDS Limberg 4 with iFAT and little ascites.			

## Data Availability

Data (ultrasound pictures) are not available due to privacy restrictions.
